# Less means more: The magnitude of synaptic plasticity along the hippocampal dorso‐ventral axis is inversely related to the expression levels of plasticity‐related neurotransmitter receptors

**DOI:** 10.1002/hipo.22816

**Published:** 2017-12-11

**Authors:** Valentyna Dubovyk, Denise Manahan‐Vaughan

**Affiliations:** ^1^ Department of Neurophysiology Medical Faculty, Ruhr University Bochum Bochum, 44780 Germany; ^2^ International Graduate School of Neuroscience Ruhr University Bochum Bochum, 44780 Germany

**Keywords:** dorsoventral axis, hippocampus, LTD, LTP, rodent, plasticity‐related receptors

## Abstract

The dorsoventral axis of the hippocampus exhibits functional differentiations with regard to (spatial Vs emotional) learning and information retention (rapid encoding Vs long‐term storage), as well as its sensitivity to neuromodulation and information received from extrahippocampal structures. The mechanisms that underlie these differentiations remain unclear. Here, we explored neurotransmitter receptor expression along the dorsoventral hippocampal axis and compared hippocampal synaptic plasticity in the CA1 region of the dorsal (DH), intermediate (IH) and ventral hippocampi (VH). We observed a very distinct gradient of expression of the N‐methyl‐D‐aspartate receptor GluN2B subunit in the Stratum radiatum (DH< IH< VH). A similar distribution gradient (DH< IH< VH) was evident in the hippocampus for GluN1, the metabotropic glutamate receptors mGlu1 and mGlu2/3, GABA_B_ and the dopamine‐D1 receptor. GABA_A_ exhibited the opposite expression relationship (DH > IH > VH). Neurotransmitter release probability was lowest in DH. Surprisingly, identical afferent stimulation conditions resulted in hippocampal synaptic plasticity that was the most robust in the DH, compared with IH and VH. These data suggest that differences in hippocampal information processing and synaptic plasticity along the dorsoventral axis may relate to specific differences in the expression of plasticity‐related neurotransmitter receptors. This gradient may support the fine‐tuning and specificity of hippocampal synaptic encoding.

## 1 INTRODUCTION

Over the past 50 years, the functional organization of the hippocampal longitudinal axis has been a focus of intensive research. Early lesion (Hughes, 1965; Moser, Moser, & Andersen, 1993; Moser, Moser, Forrest, Andersen, & Morris, 1995; Nadel, 1968), anatomical projection (Amaral and Witter, 1989; Risold and Swanson, 1996) and behavioral studies (Nadel, 1968) suggested that the dorsal (septal) hippocampus mediates spatial learning and memory, whereas the ventral (temporal) hippocampus is mainly involved in autonomic, neuroendocrine, emotional, and affective responses (Fanselow and Dong, 2010; Moser and Moser, 1998; Strange, Witter, Lein, & Moser, 2014). Moreover, a gradual functional shift from the dorsal towards the ventral hippocampal pole has been reported (Kjelstrup et al., 2008; Strange et al., 2014), whereby the intermediate hippocampus (IH) subserves a transitional role between the two hippocampal poles. This intermediate hippocampal subdivision is postulated to play a specific role in the translation of new rapid learning into efficient behavioral performance (Bast, Wilson, Witter, & Morris, 2009). More recent gene expression studies support a hippocampal differentiation of this kind (Dong, Swanson, Chen, Fanselow, & Toga, 2009; Thompson et al., 2008). Interestingly, the ventral hippocampus (VH) was shown to have a higher susceptibility to epileptic discharges (Gilbert, Racine, & Smith, 1985; Papatheodoropoulos, Moschovos, & Kostopoulos, 2005) whereas the dorsal hippocampus (DH) exhibits a higher susceptibility to ischemic damage (Ashton, Van Reempts, Haseldonckx, & Willems, 1989), which may in part depend on intrinsic dorso‐ventral differences on molecular, cellular and network levels (Doughetry, Islam, & Johnston, 2012; Kesner and Rolls, 2015; Marcelin et al., 2012).

Moreover, a functional separation of this kind along the hippocampal longitudinal axis is likely to depend on differentiated preferences for input information and information processing within the intrinsic neuronal circuits of the dorsal, intermediate, and ventral subdivisions. This may be reflected in the form of differences in synaptic plasticity, as the means through which synaptic information encoding takes place. Correspondingly, it has been reported that the VH expresses much weaker synaptic potentiation compared with its dorsal counterpart (Maggio and Segal, 2007a; Maggio, Shavit Stein, & Segal, 2015; Maruki, Izaki, Nomura, & Yamauchi, 2001; Papatheodoropoulos and Kostopoulos, 2000). In contrast, other studies have reported that the ventral hippocampal pole expresses quite strong, or even equivalent synaptic potentiation compared to potentiation elicited in the dorsal CA1 hippocampus (Kouvaros and Papatheodoropoulos, 2016). However, in case of these former reports, LTP was evoked with high‐frequency stimulation, whereas Kouvaros and Papatheodoropoulos (2016) used theta burst stimulation (TBS) protocols with varying numbers of bursts—from 1 to 8. Thus, there seems to be little consistency with regard to protocols used and LTP responses obtained. Similarly, the profile of long‐term synaptic depression in the ventral pole was reported to be either equivalent to, or of greater magnitude than, synaptic depression evoked in the dorsal CA1 (Izaki, Takita, & Nomura, 2000; Maggio and Segal, 2009a). These differences may have arisen due to differences in the hippocampal slice preparation, or afferent frequencies used to elicit synaptic plasticity. Another point that remains unclear is whether the intermediate CA1 region is able to express long‐term forms of synaptic plasticity, such as long‐term potentiation (LTP) or long‐term depression (LTD). To our knowledge, synaptic plasticity in the IH of the rat has only been examined in the dentate gyrus (Kenney and Manahan‐Vaughan, 2013a, 2013b). However, in mouse hippocampal slices, the intermediate CA1 region was shown to produce LTP of intermediate magnitude between the dorsal and ventral CA1 responses that was sustained/monitored for 60 minutes (Milior et al., 2016).

Among the most prominent plasticity‐triggering receptors are the N‐methyl‐D‐aspartate subtype of ionotropic glutamate receptors (NMDAR) (Bear and Malenka, 1994; Fox, Russell, Wang, & Christie, 2006), groups I and II metabotropic glutamate (mGlu) receptors (mGlu1, mGlu5, and mGlu2/3) (Altinbilek & Manahan‐Vaughan, 2009; Popkirov & Manahan‐Vaughan, 2011; Mukherjee & Manahan‐Vaughan, 2013), ionotropic and metabotropic GABAergic receptors (GABA_A_ and GABA_B_, respectively) (Paulsen & Moser, 1998); and dopamine receptors (D1/D5 and D2/D3) (Hansen & Manahan‐Vaughan, 2014). Very few studies report differences in binding affinity and mRNA/protein levels for the subunits of the NMDAR between the dorsal and ventral CA1 (Martens, Capito, & Wree, 1998; Pandis et al., 2006). However, analysis was never detailed and did not scrutinize the intermediate subdivision. Moreover, to our best knowledge, no information is available about the expression and distribution patterns of other plasticity‐related receptors along the hippocampal longitudinal axis in the rat. Knowledge of this kind is essential for both the interpretation and understanding of how synaptic plasticity properties may be differentiated along the hippocampal longitudinal axis.

The aim of this study was, firstly, to characterize and compare the expression and distribution of plasticity‐related proteins in the CA1 region of the dorsal, intermediate and ventral subdivisions of the hippocampus. We focused on examining subunits of the NMDAR (GluN1, GluN2A, and GluN2B), groups I and II mGlu receptors (mGlu1, mGlu5, and mGlu2/3), GABAergic receptors, and dopaminergic receptors. Our second aim was to investigate physiological properties of the CA1 neurons along the hippocampal longitudinal axis, as well as to compare the ability of the Schaffer collateral–CA1 synapses to express long‐term synaptic plasticity. We identified an expression profile for glutamatergic, GABAergic, and dopaminergic receptors that was distinct for the dorsal, intermediate, and ventral hippocampal parts. This was associated with differences in physiological properties of the CA1 neurons, along with differences in the ability of the dorsal, intermediate, and ventral hippocampal parts to express both LTP and LTD. Taken together, these data suggest that the dorsal, intermediate, and ventral hippocampal parts exhibit physiologically distinct properties, and that these distinctions are enabled at least in part, by the very different expression profiles of plasticity‐related proteins exhibited along the dorsoventral hippocampal axis. These differences may serve to explain the functional heterogeneity that is attributed to the dorsoventral axis of the hippocampus (Fanselow & Dong, 2010; Strange et al., 2014).

## 2 MATERIALS AND METHODS

### 2.1 Animals

All experiments were conducted using 6–10‐week‐old male Wistar rats (Charles River Laboratories, Sulzfeld, Germany). Animals were housed in custom‐made climatised and ventilated holding cupboards, in an animal‐housing room with a controlled 12‐h light/dark cycle. No female rats were housed in the room. Animals had free access to food and water. The study was carried out in accordance with the European Communities Council Directive of September 22, 2010 (2010/63/EU) for care of laboratory animals.

### 2.2 Immunohistochemistry

**Figure 1:**
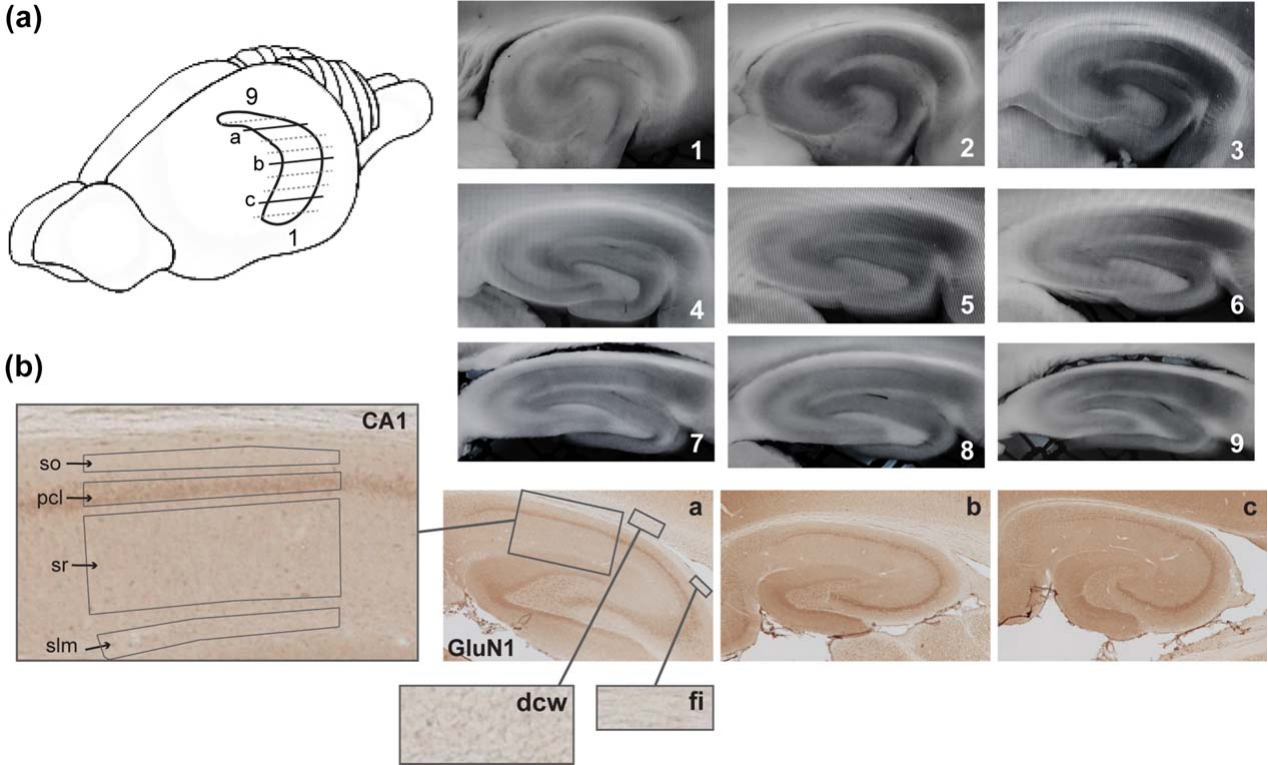
Illustration of hippocampal sectioning and resulting sections throughout its longitudinal axis. (a) A simplified schema of the rat brain showing the horizontally sectioned hippocampus. The amount of lines underrepresents the total amount of slices, which can be obtained from the hippocampus, but highlights the span of sections that could be used for electrophysiological and biochemical processing. Representative immunohistochemically‐stained transverse sections depict dorsal (a), intermediate (b), and ventral (c) hippocampal subdivisions that were used for receptor protein processing (GluN1 subunit of the NMDAR in this case). Sections 1–9 show typical slices used for electrophysiological analysis: Sections 1–3 ventral hippocampal sections; from 4 to 6 represent intermediate, and 7–9 represent dorsal hippocampal slices. (b) A close‐up view of the laminar structure of the hippocampal CA1 region and of receptor‐devoid regions (dcw, deep cerebral white matter tracts; fi, fimbria) used for background (unspecific staining) subtraction. so, Stratum oriens; pcl, pyramidal cell layer; sr, Stratum radiatum; and slm, Stratum lacunosum‐moleculare [Color figure can be viewed at wileyonlinelibrary.com]

For immohistochemical analysis, animals were euthanized with sodium pentobarbital and transcardially perfused with cold Ringer's solution + heparin (0.2%), followed by 4% paraformaldehyde (PFA) in phosphate buffered saline (PBS, 0.025 M). Brains were then removed, fixed in 4% PFA for 24 h‐, and cryoprotected in 30% sucrose in 0.1 M PBS for at least 3 days. Serial 30‐µm thick horizontal sections were collected using a freezing microtome. For each animal (*N* = 10), three horizontal sections from the most dorsal (between 3.6 and 4.1 mm posterior to bregma), middle intermediate (around 5.6 mm posterior to bregma) and most ventral hippocampal parts (between 7.1 and 7.6 mm posterior to bregma) were simultaneously used for immunohistochemical staining (Figure 1 and Supporting Information Figure S1). Free‐floating brain sections were pretreated in 0.3% H_2_O_2_ in PBS for 20 min, rinsed in PBS and then incubated with blocking solution containing 10% normal serum + 20% avidin in PBS with 0.2% Triton X‐100 (PBS‐Tx) for 90 min at room temperature. The sections were then incubated overnight at room temperature with primary antibody solutions: goat polyclonal antiNMDAɛ2 (sc‐1469, Santa Cruz Biotechnology, Santa Cruz) at 1:250 dilution, rabbit polyclonal antimGluR2/3 (ab1553, Merck Millipore, Billerica) 1:200, rabbit polyclonal antimGlu5 (ab5675, Merck Millipore, Billerica) 1:200, mouse monoclonal antiGABA_A_ receptor (mab341, Merck Millipore, Billerica) 1:400, mouse monoclonal antiGABA_B_ receptor 1 (ab55051, Abcam, Cambridge, UK) 1:250, goat polyclonal antiD1DR (sc‐1434, Santa Cruz Biotechnology, Santa Cruz) 1:100, and rabbit polyclonal antiD2R (ab1558, Merck Millipore, Billerica) 1:250 in medium containing 1% normal serum in 0.2% PBS‐Tx + 20% biotin. The sections were then washed three times for 10 min in PBS and incubated with biotinylated goat antirabbit (BA‐1000, Vector Laboratories, Burlingame), horse antimouse (BA‐2001, Vector Laboratories, Burlingame) or horse antigoat (BA‐9500, Vector Laboratories, Burlingame) antibodies at 1:500 dilution in 1% normal serum in 0.1% PBS‐Tx for 90 min at room temperature, respectively. Afterwards, sections were washed three times for 10 min in PBS and incubated for 90 min at room temperature with an avidin‐biotin complex (ABC) kit (PK‐6100, Vector Laboratories, Burlingame) in 1% normal serum in 0.1% PBS‐Tx. For other receptors an additional amplification step with biotinylated tyramide for 20 min was performed. Here, the sections were incubated for 5 days at 4 °C with primary antibody solutions: mouse monoclonal antiNMDAR1 (556308, PharMingen, Becton, Dickinson and Company, Frankline Lakes) at 1:200 dilution, rabbit polyclonal antiNMDAɛ1 (sc‐9056, Santa Cruz Biotechnology, Santa Cruz) 1:750, and rabbit polyclonal antimGlu1 (ab82211, Abcam, Cambridge, UK) 1:400 in a dilution medium containing 1% BSA in 0.2% Triton X‐100 in tris‐buffered saline. The staining was performed as described above with a few variations: PBS was replaced with tris‐buffered saline, normal serum + PBS‐Tx with bovine serum albumin + Triton X‐100 in tris‐buffered saline, and one ABC reaction with two for 30 min each with amplification step in between. Here, sections were incubated with 10 µL b‐tyramide + 10 µL 0.01% H_2_O_2_ in 1000 µL of tris‐buffered saline for 20 min. Finally, the sections were washed in PBS and treated with diaminobenzidine (DAB) and 0.01% H_2_O_2_ for ∼10 min.

Every 12th section throughout the whole hippocampus was used for Nissl staining with 0.1% Cresylviolet. The following regions of interest (ROIs) were scrutinized: Stratum oriens (so) of the CA1; pyramidal cell layer (pcl) of the CA1; Stratum radiatum (sr) of the CA1; Stratum lacunosum‐moleculare (slm) of the CA1 on sections taken from the dorsal, intermediate and ventral hippocampal subdivisions (Figure 1). Photomicrographs of stained sections were acquired with a light microscope (Leica DMR, Wetzlar, Germany), equipped with a digital camera (MBF Bioscience, Vermont) and stored in TIFF format. The hippocampal ROIs were analyzed at 2.5× lens magnification. The digital high resolution pictures were obtained using Neurolucida software (MBF Bioscience, Vermont) and quantified using open‐source ImageJ software (National Institute of Health, USA). Given that images were acquired with a red, green, blue camera, the “Color Deconvolution” plugin in ImageJ was used to deconvolve the color information and to convert images to eight‐bit format, thus, increasing the dynamic range of color representation (Jacqui Ross, 2014). As a next step, the background staining was subtracted from each image. In the dorsal hippocampal sections, receptor‐devoid tissue appears in a form of fimbria (fi) and deep cerebral white matter tracts (dcw), in the intermediate sections this comprises fi, external, and internal capsules (ec & ic) and the superior thalamic radiation (str), and in the ventral sections this comprises fi and ic. Therefore, background values from fi and dcw for the dorsal hippocampal sections; fi, ic, and str for the intermediate hippocampal sections; and fi and ic for the ventral hippocampal sections were averaged and then this averaged value was subtracted from corresponding images. Finally, R software was used to scale data from several independent stainings/plates using generalized residual sum of squares algorithm to account for batch effects of staining intensities (Kreutz et al., 2007; von der Heyde et al., 2014).

### 2.3 Immunoblotting

Two protein biochemical methods were used as specificity controls for immunohistochemical experiments (Supporting Information Figure S2):

1. direct immunoblot analysis from whole tissue lysate or,
2. immunoprecipitation that was followed by immunoblotting.

For immunoblotting experiments, brains were rapidly removed followed by whole hippocampus dissection. The tissue were then homogenized in 20 mM Tris–HCl buffer (pH 7.4) containing 10% sucrose followed by centrifugation at 14,000 rpm for 30 min at 4 °C in 20 mM Tris–HCl buffer supplemented with protease inhibitor. The supernatant was removed and the pellet was resuspended in 20 mM Tris–HCl buffer supplemented with protease inhibitor. The protein concentration of the samples was determined with the Bradford assay (500‐0006, Bio‐Rad Laboratories, München, Germany). Protein samples were separated in 8% sodium dodecyl sulfate polyacrylamide gels, which then were electroblotted onto polyvinylidene difluoride membranes. Membranes were blocked for 1 h‐ (5% nonfat dry milk, 0.1% Tween 20 in TBS) at room temperature. Blots were then incubated overnight at 4 °C with primary antibodies: mouse monoclonal anti‐NMDAR1 (556308, PharMingen, Becton, Dickinson and Company, Frankline Lakes) at 1:750 dilution, rabbit polyclonal anti‐NMDARɛ1 (sc‐9056, Santa Cruz Biotechnology, Santa Cruz) 1:250, goat polyclonal anti‐NMDAɛ2 (sc‐1469, Santa Cruz Biotechnology, Santa Cruz) 1:500, rabbit polyclonal anti‐mGlu1 (ab82211, Abcam, Cambridge, UK) 1:1,000, rabbit polyclonal anti‐mGlu2/3 (ab1553, Merck Millipore, Billerica) 1:500, rabbit polyclonal anti‐mGlu5 (ab5675, Merck Millipore, Billerica) 1:4,000, mouse monoclonal anti‐GABA_A_ receptor (mab341, Merck Millipore, Billerica) 1:500, mouse monoclonal anti‐GABA_B_ receptor 1 (ab55051, Abcam, Cambridge, UK) 1:500, goat polyclonal anti‐D1DR (sc‐1434, Santa Cruz Biotechnology, Santa Cruz) 1:250, and rabbit polyclonal anti‐D2R (ab1558, Merck Millipore, Billerica) 1:1,000. Membranes were washed 3 times in 0.1% Tween 20 in TBS and incubated with antimouse and antirabbit horseradish peroxidase linked IgG (NA931V and NA934V, GE Healthcare, Freiburg, Germany) as secondary antibodies at 1:5,000 to 1:20,000 dilution ranges for 90 min at room temperature. Protein bands were visualized using an enhanced chemiluminescence reagent (ECL), Pierce ECL Plus, or ECL Prime, on X‐ray films or CCD camera.

For immunoprecipitation experiments, a tissue lysate in a volume of 200 µg was filled up to 400 µL of total volume with sample buffer. 25 µL of 50% Protein A Sepharose (PAS) beads and 4 µL of primary antibodies (anti‐NMDAɛ1 or anti‐NMDAɛ2) were added. Immunocomplexes were captured through overnight incubation at 4 °C. PAS beads were then briefly centrifuged (20–30 s) and rinsed in sample buffer. The procedure was repeated three times. After the last rinsing, 25–30 µL of liquid were left on top of the Sepharose beads and the same amount of 2× Laemmli buffer was added. From here on, immunoprecipitation was followed by immunoblotting as described earlier.

### 2.4 *In vitro* electrophysiology

Brains were dissected in ice‐cold (1–4 °C), oxygenated artificial cerebrospinal fluid (aCSF) containing (in mM: 124 NaCl; 4.9 KCl; 1.2 NaH_2_PO_4_; 1.3 MgSO_4_; 2.5 CaCl_2_; 25.6 NaHCO_3_; and 10 D‐glucose; pH 7.4). The two hippocampi were isolated and then sectioned into 400‐µm thick slices using a vibratome (VT 1000S, Leica, Nussloch, Germany). Specifically, transverse slices of the longitudinal axis of the hippocampus were prepared as shown on Figure 1. Here, slices from the dorsal, intermediate and ventral hippocampal subdivisions were used. Slices were incubated for 15 min at ∼35 °C, and then placed on a nylon net in separate submerged recording chambers for at least 1 h prior to any recordings. Slices were continuously perfused at a constant flow rate of 2 mL/min with oxygenated aCSF at 32–33 °C. Field recordings were made with a metal electrode (platinum/tungsten core, impedance: 0.5 MΩ; Thomas Recording, Gießen, Germany) positioned in the sr of the CA1 region. Stimulation was delivered through a bipolar electrode (Fredrick Haer, Bowdowinham, ME) placed in the Schaffer collaterals. Test‐pulse stimuli of 0.2 ms duration were applied at 0.025 Hz to evoke field excitatory postsynaptic potentials (fEPSPs) with a sample rate of 10,000 Hz. For each time‐point, five responses were averaged across a 5 min interval. An input–output (I/O) curve was obtained prior to commencing experiments (stimulation range: 60–600 µA in 10 steps) and a test‐pulse stimulation strength was used that evoked a fEPSP that comprised 50% of the I/O maximum. Following 40 min of baseline recordings, LTP was induced by two trains of TBS delivered 10 s apart (each train was composed of 10 bursts of four pulses each, at 100 Hz) at a frequency of 4 Hz. LTD was generated by a low‐frequency burst stimulation protocol (LFBS), which consisted of two trains of burst stimulation (each train composed of 10 bursts of four pulses each, at 250 Hz) delivered 20 s apart at the frequency of 25 Hz. LFBS was used in preference to the standard low‐frequency stimulation, as stimulation patterns composed of 250 Hz bursts are within the neural firing and ripple frequencies of hippocampal CA1 region in awake rats (Suzuki and Smith, 1988; Ylinen et al., 1995). Moreover, such a firing pattern may play a role in physiological inhibitory regulation within the hippocampus and was shown to be more effective in triggering the ventral hippocampal LTD (Izaki et al., 2000).

Paired‐pulse responses were examined by applying afferent stimulation in the form of two pulses of equal intensity and duration (0.2 ms) at interpulse intervals (IPIs) of 10, 20, 25, 50, and 100 ms. Individual pairs of stimuli were delivered at 40 s intervals, and individual IPI blocks of stimulation were delivered at 5 min intervals. Five stimulation pairs at each IPI were averaged and used for the analysis.

For the whole‐cell current‐clamp recordings, hippocampal sections from the dorsal, intermediate, and ventral subdivisions were continuously perfused in heated (∼32 °C) oxygenated aCSF (as described earlier). Our aCSF solution did not contain any agents that would influence the triggering fast EPSPs/IPSPs (e.g., picrotoxin). Pyramidal neurons in the middle of the proximodistal axis of the CA1 region were visualized at 40× magnification using an Olympus BX51WI microscope and an infrared video camera (TILL Photonics, Gräfelfing, Germany). Recording patch electrodes (6–9 MΩ) were pulled from borosilicate glass with an external diameter of 1.5 mm using a Flaming/Brown micropipette puller (P‐1000, Sutter Instruments, CA). Electrodes were filled with an intracellular solution containing (in mM: 97.5 K‐gluconate; 32.5 KCl; 10 HEPES; 1 MgCl_2_; 4 Na_2_ATP; 5 EGTA; pH 7.3). Whole‐cell current‐clamp recordings were performed on the soma of the CA1 pyramidal neurons, without a correction for liquid junction potentials, using a HEKA EPC10 amplifier and PATCHMASTER data acquisition software. Signals were low‐pass filtered at 2.9 kHz and digitized at 10 kHz. Data were analyzed in an off‐line mode in FITMASTER program.

The somatic input resistance (R_in_) was measured as the slope of the voltage‐current plot generated in a response to hyperpolarizing and depolarizing current injections (–80 to + 20 pA, steps of 20 pA). The membrane time constant was calculated as the slow component of an exponential fit of the averaged voltage decay in response to a hyperpolarizing current injection (–40 pA, 600 ms). Single action potentials were analyzed for action potential threshold (current and voltage), action potential amplitude, action potential half‐width and afterhyperpolarization (AHP). Threshold (current) was defined as the current needed to induce an action potential. Threshold (voltage) was determined as the membrane voltage by reaching which an action potential is generated and was measured from the resting membrane potential (RMP). Action potential amplitude was measured as the voltage difference from threshold to peak, with the half‐width measured at half this distance. AHP was determined as the voltage difference from the RMP to the peak of an undershoot. Firing frequency was calculated by averaging the instantaneous firing frequency of action potentials in a response to depolarizing current injections ranging from 50 to 400 pA.

### 2.5 Data analysis

For all experiments “N” corresponds to the number of animals while “n” corresponds to the number of hippocampal slices, or cells. Data obtained in the immunohistochemical experiments were statistically analyzed using a factorial analysis of variance (ANOVA) followed by a Duncan's *post hoc* test. All significant differences were defined as *p* < .05 or .01. Values are expressed as mean values ± the *SEM*. Paired‐pulse responses were quantified as the ratio of the second pulse‐evoked fEPSP to the first one. All electrophysiological data were statistically analyzed using a factorial ANOVA with repeated measures followed by a Fisher's *post hoc* test, or a *t*‐test when applicable. Since the slope of fEPSPs at the synapses strongly correlated with changes of the amplitude, mean slope values are exclusively presented. Summary graphs are shown as mean ± *SEM*. Data obtained in the patch‐clamp experiments were statistically analyzed using a one‐way ANOVA followed by Tukey HSD test. In case of the firing frequency, a factorial ANOVA with repeated measures followed by a Fisher's *post hoc* test was used.

## 3 RESULTS

### 3.1 GluN1/GluN2B receptor expression is highest in the ventral CA1. GluN1/GluN2A receptors are equally expressed across the longitudinal axis of the hippocampus

An immunohistochemical approach was used to compare the expression and distribution profiles of glutamatergic, GABAergic and dopaminergic receptors across the longitudinal hippocampal axis. The CA1 region of the DH, IH, and VH were compared. In each hippocampal section, we assessed receptor protein levels in a layer‐specific manner, including basal dendrites, cell soma and apical dendrites of the CA1 cells.

**Figure 2:**
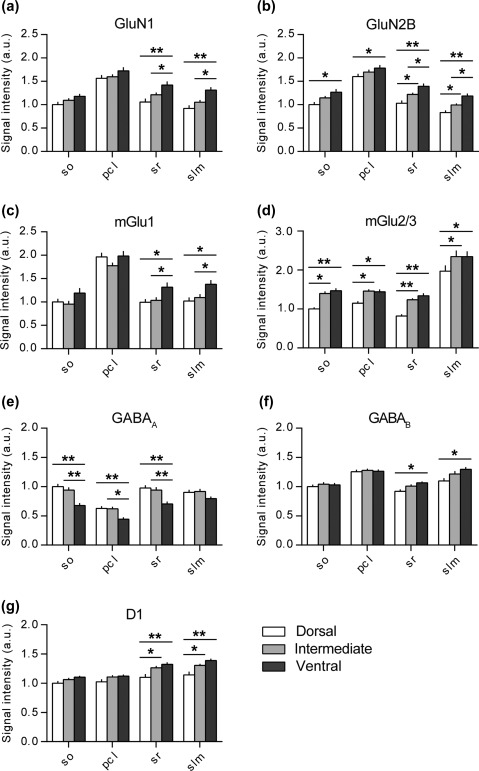
Dorsal, intermediate and ventral parts of the CA1 region express different amounts of plasticity‐related receptors. Bar graphs illustrate the relative change in protein expression of the NMDAR subunits (GluN1 and GluN2B), group I and II mGlu receptors (mGlu1 and mGlu2/3), GABAergic receptors (GABA_A_ and GABA_B_) and dopamine D1 receptors across the somato‐dendritic and longitudinal axes of the CA1 region. (a) GluN1 protein density levels were the highest in the ventral apical dendrites. (b) Similarly, the GluN2B levels were the highest in the ventral CA1 across its entire somato‐dendritic axis. Here, the protein expression in the apical dendrites showed gradual and significant increase from the dorsal towards the ventral pole. (c) Again, the mGlu1 protein density levels were the highest in the ventral apical dendrites of the CA1 region. (d) MGlu2/3 protein levels were the lowest in the dorsal CA1 and comparable in the intermediate and ventral domains. (e) GABA_A_ protein expression was the lowest in the ventral CA1 in most layers and did not differ between the dorsal and intermediate parts. (f) GABA_B_ levels were significantly higher in the ventral apical dendrites and were equally expressed in the cell soma and basal dendritic layers. (g) D1 protein levels were significantly higher in the ventro‐intermediate apical dendrites as opposed to their dorsal counterpart. Values expressed in arbitrary units. Error bars indicate *SEM*. *p < .05 or **p < .01. pcl, pyramidal cell layer; so, Stratum oriens; sr, Stratum radiatum; slm, Stratum lacunosum‐moleculare

GluN1 protein density levels were significantly higher in two regions of the ventral CA1 (*N* = 10) compared with the dorsal and intermediate subdivisions (multifactorial ANOVA: *F*_[2,204]_ = 19.627, *p* < .001) (Figure 2a). Specifically, significant effects were found in the sr and slm of the CA1 region (Duncan's *post hoc* test for sr: DH vs. VH, *p* < .001; IH vs. VH, *p* = .02 and slm: DH vs. VH, *p* < .001; IH vs. VH, *p* = .007). No differences could be observed between the dorsal and intermediate CA1.

The GluN2A subunit of the NMDAR was evenly expressed across all layers of the dorsal, intermediate and ventral CA1 region (multifactorial ANOVA: *p* = .35, Supporting Information Figure S3A).

Similar to the GluN1 expression profile, GluN2B levels were significantly higher at the ventral pole (multifactorial ANOVA: *F*_(2,228)_ = 29.216, *p* < .001) (Figure 2b). Overall, GluN2B expression was lowest in the so (DH vs. VH, *p* = .001), pcl (DH vs. VH, *p* = .02), sr (DH vs. VH, *p* < .001) and slm (DH vs. VH, *p* < .001) of the dorsal CA1 compared with its ventral counterpart. In case of the apical dendrites, namely sr (DH vs. IH, *p* = .02; IH vs. VH, *p* = .02) and slm (DH vs. IH, *p* = .03; IH vs. VH, *p* = .02), a significant gradual increase from the dorsal toward the ventral pole was evident.

### 3.2 MGlu1 expression is the highest in the ventral CA1, while mGlu5 levels are equivalent across the longitudinal axis. MGlu2/3 expression is the lowest in the dorsal CA1

Assessment of mGlu1 receptor expression revealed significantly higher protein levels in the apical dendritic layers compared with the other neuronal/synaptic subcompartments (multifactorial ANOVA: *F*_[2,208]_ = 10.421, *p* < .001). Here, an expression gradient was found, whereby the VH exhibited the highest levels. Specific effects were found in the sr (VH vs. DH, *p* = .01; VH vs. IH, *p* = .03) and slm (VH vs. DH, *p* = .007; VH vs. IH, *p* = .03) (Figure 2C). No differences were detected between the dorsal and intermediate CA1.

In case of the mGlu5 receptor, there were no differences in the protein levels between any of the hippocampal subdivisions or layers of the CA1 region (multifactorial ANOVA: *p* = .18, Supporting Information Figure S3B).

For mGlu2/3, levels were the lowest in the dorsal CA1 as opposed to its intermediate and ventral counterparts (multifactorial ANOVA: *F*_[2,208]_ = 27.7, *p* < .001) (Figure 2d). Here, every layer of the CA1 region followed the same pattern. (Duncan's *post hoc* test for so: DH vs. IH, *p* = .002; DH vs. VH, *p* < .001, pcl: DH vs. IH, *p* = .02; DH vs. VH, *p* = .02, sr: DH vs. IH, *p* < .001; DH vs. VH, *p* < .001 and slm: DH vs. IH, *p* = .001; DH vs. VH, *p* = .002. No differences were detected between the intermediate and ventral CA1.

### 3.3 GABAA expression is the lowest in the ventral CA1, whereas GABAB expression exhibits a heterogeneous profile

GABA_A_ protein levels showed an opposite expression to that exhibited by the excitatory plasticity‐related receptors (multifactorial ANOVA: *F*_[2,212]_ = 32.36, *p* < .001) (Figure 2E). Here, the layers of the ventral CA1, namely so (VH vs. DH, *p* < .001; VH vs. IH, *p* < .001), pcl (VH vs. DH, *p* = .003; VH vs. IH, *p* = .003) and sr (VH vs. DH, *p* < .001; VH vs. IH, *p* < .001) expressed the lowest levels of the GABA_A_ receptor compared with the dorsal and intermediate counterparts. No differences were found between the dorsal and intermediate CA1.

GABA_B_ protein levels were evenly expressed across the basal dendrites (so) and cell somas (pcl) of the dorsal, intermediate and ventral CA1 region (Figure 2F). Significantly higher receptor levels (multifactorial ANOVA: *F*_[2,228]_ = 7.33, *p* < .001) were found across the apical dendrites of the ventral CA1 compared with the dorsal part, in case of sr (VH vs. DH, *p* = .01) and slm (VH vs. DH, *p* < .001).

### 3.4 D1 expression is the lowest in the dorsal CA1, while D2 is comparable across the dorsoventral axis

Similarly to the expression pattern of the glutamatergic receptors, dopamine D1 receptor levels were significantly lower in the apical dendrites of the dorsal CA1 as compared with the ventro‐intermediate two‐thirds (multifactorial ANOVA: *F*_[2,224]_ = 21.83, *p* < .001) (Figure 2g). Here, the difference was found in both sr (DH vs. VH, *p* < .001; DH vs. IH, *p* = .003) and slm (DH vs. VH, *p* < .001; DH vs. IH, *p* = .002) layers. Intermediate and ventral CA1 showed equivalent D1 expression across all layers examined.

Dopamine D2 protein levels were equivalently expressed across all layers of the dorsal, intermediate, and ventral CA1 region (multifactorial ANOVA: *p* = .14, Supporting Information Figure S3C).

### 3.5 Somatic excitability properties of the CA1 pyramidal neurons are similar between the dorsal and intermediate parts, but distinct from the ventral ones

The differences in receptor expression that we detected along the dorsoventral hippocampal axis, suggest that neurons and synapses of these subdivisions may exhibit different physiological profiles. For this reason, we then examined whether CA1 pyramidal neurons exhibit differences when the neurons of the dorsal (*N* = 6, *n* = 13), intermediate (*N* = 6, *n* = 15) and ventral (*N* = 6, *n* = 21) subdivisions were compared.

**Figure 3:**
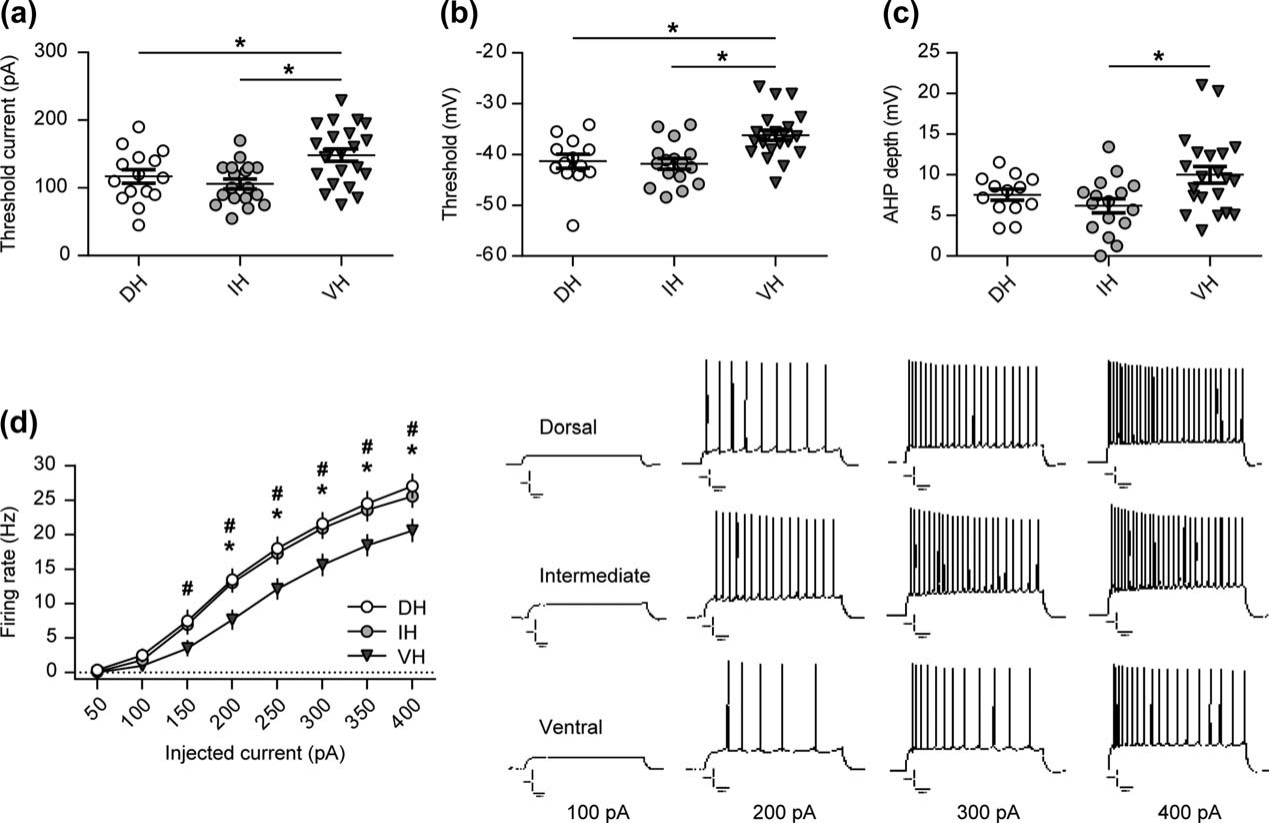
Intrinsic excitability and firing properties of the ventral CA1 neurons are distinct from those in the dorsal and intermediate domains. (a) Patch‐clamp recordings from the single cells revealed that ventral CA1 neurons require stronger afferent stimulation to elicit action potential firing. It is supported with significantly more depolarized threshold for the action potential induction in the ventral pyramidal cells compared with the dorso‐intermediate ones (b). (c) Additionally, ventral CA1 pyramidal neurons show significantly deeper after hyperpolarization (AHP) as opposed to the other two domains. (d) Application of depolarizing currents (from 50 to 400 pA) revealed a difference in spike frequency patterns, where the firing rate of ventral CA1 neurons was significantly lower in comparison to both, dorsal and intermediate ones. Current clamp recordings of repetitive firing evoked by current injections (100, 200, 300, and 400 pA) to hippocampal CA1 neurons are shown on the right. Error bars indicate *SEM*. */#*p* < .05, where * indicates significant difference between ventral and intermediate slice groups and # indicates significant difference between ventral and dorsal slice groups. Scale bars: 10 mV, 100 ms

We found that the firing frequency‐to‐current injection (*F–I*) relationship was almost identical for dorsal and intermediate hippocampal CA1 cells, while ventral neurons exhibited an *F–I* relationship that was significantly shifted to the right (repeated measures ANOVA: *F*_[2,46]_ = 4.44, *p* = .01) (Figure 3d). In the stimulation intensity range of 200 pA through 400 pA, the firing frequency of action potentials in the ventral CA1 neurons was significantly lower compared with both other groups (Fisher's *post hoc* test, *p* < .01). That is, higher current injections are needed for the ventral neurons to elicit equivalent firing to the dorsal and intermediate neurons. Similarly, the ventral CA1 neurons required higher current injections to reach the threshold for the generation of the first action potential (one‐way ANOVA: *F*_[2,46]_ = 8.25, *p* < .001) (Figure 3a; Table 1).

**Table 1:** Comparison of membrane properties of pyramidal neurons across longitudinal axis of the CA1 region

Membrane properties	Dorsal	Intermediate	Ventral	ANOVA *F* =; *p* =
RMP (mV)	−61.14 ± 0.71 (13)	−62.5 ± 0.83 (15)	−62.48 ± 0.64 (21)	*F*_(2,46)_ = 0.99; *p* = .37
R_in_ (MΩ)	63 ± 3.61 (13)	74.43 ± 4.75 (15)	65.59 ± 3.83 (21)	*F*_(2,46)_ = 1.88; *p* = .16
Membrane time constant (ms)	9.88 ± 0.66 (13)	13.22 ± 1.33 (15)	11.87 ± 0.87 (21)	*F*_(2,46)_ = 2.33; *p* = .1
AP amplitude (mV)	97.89 ± 1.79 (13)	98.31 ± 1.42 (15)	92.96 ± 1.61 (21)	*F*_(2,46)_ = 3.62; ***p* = .03**
AP half‐width (ms)	0.79 ± 0.02 (13)	0.82 ± 0.02 (15)	0.83 ± 0.01 (21)	*F*_(2,46)_ = 0.9; *p* = .41
Current threshold (pA)	115 ± 10.68 (13)	101.33 ± 8.14 (15)	151.14 ± 9.09 (21)	*F*_(2,46)_ = 8.25; ***p* < .001**
Voltage threshold (mV)	−41.33 ± 1.36 (13)	−41.76 ± 1.14 (15)	−36.21 ± 1.01 (21)	*F*_(2,46)_ = 7.96; ***p* = .001**
AHP depth (mV)	7.55 ± 0.67 (13)	6.3 ± 0.92 (15)	10 ± 1.02 (21)	*F*_(2,46)_ = 4.2; ***p* = .02**

The table describes the mean ± *SEM* values. *N* values represent the amount of cells and are indicated in parentheses. Bold font highlights significant differences.

Differences in the *F–I* relationship could not be accounted for by differences in RMP (one‐way ANOVA: *F*_[2,46]_ = 0.99, *p* = .37) or somatic R_in_ (one‐way ANOVA: *F*_[2,46]_ = 1.88, *p* = .16) as they were comparable in all three groups (Table 1). However, they seem, at least in part, to depend on differences in threshold (voltage) between the ventral, dorsal, and intermediate neurons, whereby the ventral CA1 pyramidal cells exhibited significantly more depolarized values than the dorsal or intermediate ones (one‐way ANOVA: *F*_[2,46]_ = 7.96, *p* = .001) (Figure 3b; Table 1). Additionally, the *F‐I* differences might arise from significantly deeper AHP in the ventral CA1 neurons as opposed to the dorsal and intermediate neurons (one‐way ANOVA: *F*_[2,46]_ = 4.2, *p* = .02) (Figure 3c; Table 1).

With regard to the other parameters measured, we also found a significant difference in the action potential amplitude between the ventral cells and neurons from the other two subdivisions (one‐way ANOVA: *F*_(2,46)_ = 3.62, *p* = .03), but no change in the action potential half‐width (one‐way ANOVA: *F*_(2,46)_ = 0.9, *p* = .41), or in the membrane time constant (one‐way ANOVA: *F*_(2,46)_ = 2.33, *p* = .1) (Table 1).

### 3.6 LTP and LTD profiles differ at the Schaffer collateral–CA1 synapses across the hippocampal longitudinal axis

To ascertain if differences could be identified on the level of synaptic plasticity between the dorsal, intermediate, and ventral parts of the CA1 region, two forms of long‐term synaptic plasticity, namely LTP and LTD were examined.

**Figure 4:**
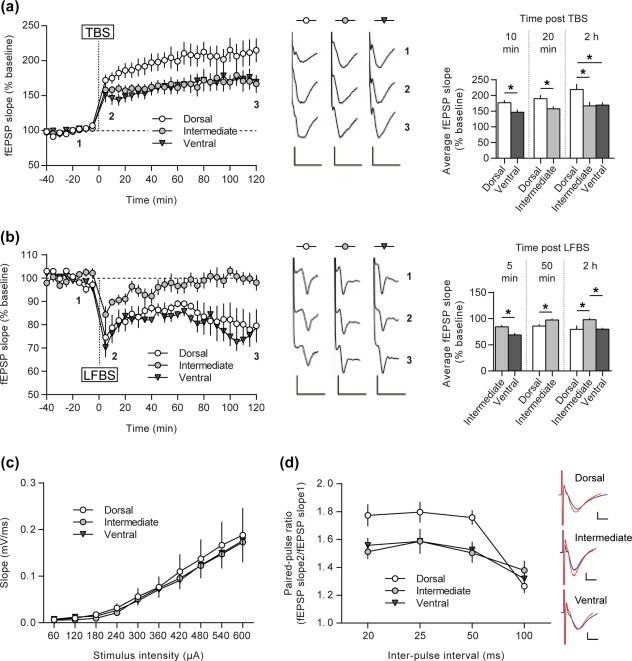
Dorsal, intermediate, and ventral parts of the CA1 differ in their ability to express LTP and LTD forms of synaptic plasticity. (a) TBS‐induced LTP persisted for over 2 h. LTPs of intermediate and ventral groups were comparable, but significantly lower in magnitude as opposed to the dorsal CA1 group. Induction (10‐ and 20‐min postTBS) as well as maintenance phases of LTP (2‐h postTBS) were significantly higher in the dorsal CA1. (b) LFBS induced equivalent LTD in both dorsal and ventral slice groups and only STD in the intermediate CA1. The initial depression was significantly different between the intermediate and ventral domains (5‐min postLFBS). Fifty minutes after the onset of depression in the intermediate group it has declined to the baseline level and remained there as shown 2‐h‐postLFBS. Representative fEPSP traces at the indicated time‐points in the graph, are shown. (c) I/O relationship does not differ between any of the hippocampal subdivisions. (d) Paired‐pulse responses facilitate greater for the DH at 20–50 ms intervals as opposed to the ventro‐intermediate two‐thirds. Typical fEPSP traces in response to paired‐pulse stimulation with a 20 ms interval are shown on the right side. Here, the blue line is response to the first pulse, while the red line is response to the second pulse. Error bars indicate *SEM*. *p < .05. Scale bars: 0.5 mV, 10 ms for LTP, 0.25 mV, 5 ms for LTD and 0.5 mV, 1 ms for paired‐pulse responses [Color figure can be viewed at wileyonlinelibrary.com]

In order to elicit LTP, we applied TBS to hippocampal slices from the dorsal (*N* = 7, *n* = 9), intermediate (*N* = 7, *n* = 8) and ventral (*N* = 10, *n* = 12) hippocampal subdivisions. In all three groups, LTP was triggered at Schaffer collateral–CA1 synapses of all subdivisions that persisted for at least 2 h (*t*‐test, DH: 199.87 ± 2.5% of baseline, *t* = 2‐h postTBS, *p* < .001; IH: 165.38 ± 10.56% of baseline, *t* = 2‐h postTBS, *p* < .001; VH: 162.85 ± 9.49% of baseline, *t* = 2‐h postTBS, *p* < .001) (Figure 4a). However, LTP was significantly different when responses in the different subdivisions were compared (repeated measures ANOVA: *F*_[2,27]_ = 3.81, *p* = .03). In dorsal slices the magnitude of LTP induction was significantly higher than in ventral slices 10 min after the onset of potentiation (*t*‐test, DH: 176.54 ± 8.68% vs. VH: 146.3 ± 9.24%, *p* = .03). Similarly, the induction phase of LTP was significantly stronger in dorsal slices compared with intermediate ones, but 20 min after the onset of potentiation (*t*‐test, DH: 189.55 ± 11.36% vs. IH: 157.21 ± 7.89%, *p* = .03). Two hours after TBS, the magnitude of fEPSP in dorsal slices was still significantly different from intermediate and ventral ones (*t*‐test, DH: 218.43 ± 18.52% vs. IH: 166.9 ± 12.72%, *p* = .04; DH: 218.43 ± 18.52% vs. VH: 169.42 ± 8.78%, *p* = .01).

In summary, LTP was stronger in the dorsal pole and was equivalent in the intermediate and ventral hippocampal subdivisions.

To elicit LTD, we applied LFBS to the dorsal (*N* = 5, *n* = 5), intermediate (*N* = 8, *n* = 8) and ventral (*N* = 6, *n* = 6) hippocampal CA1 regions. LFBS induced significant synaptic depression in all three groups (*t*‐test, DH: 83.21 ± 0.76% of baseline, *t* = 2‐h postLFBS, *p* < .001; IH: 96.4 ± 0.89% of baseline, *t* = 2‐h postLFBS, *p* = .03; VH: 80.11 ± 0.89% of baseline, *t* = 2‐h postLFBS, *p* < .001) (Figure 4B). However, the magnitude of initial depression was statistically different between the ventral and intermediate groups, whereupon the intermediate CA1 showed weaker synaptic depression (*t*‐test, VH: 68.96 ± 3.66% vs. IH: 84.33 ± 3.55%, *p* = .01, *t* = 5 min). Comparison of fEPSPs 5 min after LFBS did not reveal any difference in synaptic depression between the dorsal and intermediate subdivisions (*t*‐test, DH: 74.55 ± 6.36% vs. IH: 84.33 ± 3.55%, *p* = .17). However, starting from 50 min after LFBS onwards, synaptic depression in the intermediate CA1 became significantly weaker than depression elicited in the dorsal CA1 (Fisher's *post hoc* test, *p* = .04, *t* = 50 min; *p* = .04, *t* = 70 min; *p* = .001, *t* = 90 min; *p* < .001, *t* = 110 min). Specifically, the intermediate CA1 potentials decayed back to pre LFBS levels (*t*‐test, IH: 99.05 ± 0.47% of baseline, *t* = 50–120‐min postLFBS, *p* = .27). Similarly, two hours after LFBS, synaptic depression in ventral slices was still significantly better than that elicited in the intermediate CA1 (*t*‐test, VH: 79.81 ± 2.64% vs. IH: 98.07 ± 3.3%, *p* < .001).

In summary, LTD was stronger in the dorsal and ventral poles, whereas the same afferent stimulation protocol resulted in STD in the intermediate subdivision.

The I/O curves for the dorsal, intermediate and ventral hippocampus indicated that a similar stimulus‐response relationship was present in all hippocampal subdivisions (repeated measures ANOVA: *F*_[2,378]_ = 0.14, *p* = .86, Figure 4C).

These findings show that all three domains of the longitudinal CA1 axis express LTP that differs in the magnitude of potentiation, with the ventral and intermediate CA1 showing the weakest and the dorsal CA1 exhibiting the strongest LTP.

### 3.7 Neurotransmitter release probability is the lowest in the dorsal hippocampus

To estimate if neurotransmitter release differs along the dorsoventral hippocampal axis, we then assessed neurotransmitter release probability at Schaffer collateral–CA1 synapses with the help of a widely used proxy method known as the paired‐pulse response paradigm (Dobrunz & Stevens, 1997; Regehr, 2012). IPIs of 20, 25, 50, and 100 ms were used. Here, paired‐pulse facilitation in the dorsal CA1 (*N* = 10, *n* = 16) was significantly higher than in the intermediate (*N* = 8, *n* = 11) or ventral (*N* = 11, *n* =15) subdivisions at the 20 ms (Fisher's *post hoc* test, DH vs. IH, *p* = .002; DH vs. VH, *p* = .006), 25 ms (DH vs. IH, *p* = .01; DH vs. VH, *p* = .008) and 50 ms (DH vs. IH, *p* = .003; DH vs. VH, *p* = .003) IPI. No differences were detected at the 100 ms IPI between any of the hippocampal parts (Figure 4d). These findings suggest that the intermediate‐ventral two thirds of the hippocampal axis exhibit a higher neurotransmitter release probability compared with the dorsal third.

## 4 DISCUSSION

This study describes a detailed characterization of the expression of plasticity‐related receptors across the dorsal, intermediate and ventral subdivisions of the hippocampus. In addition we conducted a physiological comparison of neuronal properties and synaptic plasticity in these subdivisions.

Our key findings are the following:

1. Plasticity‐related receptors display distinct and subcompartment‐specific expression profiles across the dorsal, intermediate and ventral parts of the CA1 region, whereby expression is typically lowest in the dorsal and highest in the ventral subdivisions, with the GABA a receptor expression comprising the sole exception to this pattern;
2. Pyramidal neurons of the ventral CA1 differ in their intrinsic physiological characteristics and firing properties compared with neurons of the dorsal and intermediate subdivisions, showing reduced firing frequency, a more depolarized (voltage) threshold and a deeper AHP;
3. Synaptic plasticity profiles expressed by the Schaffer collateral–CA1 synapses also differ between these three domains, whereby LTP exhibits the highest magnitude in the dorsal compared with intermediate and ventral subdivisions. In contrast, LTD is equivalent in the dorsal and ventral subdivisions, whereas the intermediate subdivision expresses STD.

These findings offer new mechanistic insights as to the physiological basis of differences in information encoding across the dorsoventral axis of the hippocampus.

### 4.1 Expression profiles of the plasticity‐related receptors and their relation to neuronal excitability

We observed a gradual increase in the expression of the GluN2B‐containing NMDARs from the dorsal towards the VH. Together with comparable expression of the GluN2A‐containing NMDARs detected across the dorsoventral axis of the CA1 region, this results in a distinct ratio for the expression of GluN2A‐ and GluN2B‐containing NMDARs within the dorsal, intermediate and ventral subdivisions. This finding aligns well with the work of Pandis et al (2006), that reported significantly longer NMDAR‐mediated currents in the ventral, but not dorsal Schaffer collateral–CA1 synapse: GluN2B‐containing NMDARs exhibit channel opening times that last approximately three times longer than that of GluN2A‐containing receptors (Wyllie, Livesey, & Hardingham, 2013).

We found higher dopamine D1 receptor levels in the ventral and intermediate CA1 compared with the dorsal subdivision. D2 receptors were equally distributed. Thus, stronger D1‐mediated effects are likely to occur in the ventro‐intermediate hippocampal two‐thirds. This may relate to the possibility that these parts of the hippocampus contribute to social and emotional information processing (Segal, Richter‐Levin, & Maggio, 2010; Strange et al., 2014).

In contrast to all other receptors scrutinized here, that generally showed an expression gradient whereby the VH typically showed highest and the dorsal lowest expression levels, we found that the VH expresses lower levels of GABA_A_ receptors compared with the dorsal and intermediate CA1 parts. This would suggest that inhibitory control should be lower at the ventral pole: a possibility that is supported by the work of Sotiriou, Papatheodoropoulos, and Angelatou (2005), who reported a weaker efficacy of GABA_A_‐mediated inhibition in the ventral subdivision compared with other subdivisions of the dorsoventral hippocampal axis. Thus, lower GABA_A_ levels in the ventral CA1 may serve to counterbalance the changes in GluN2A/GluN2B ratio in this pole compared with the dorsal pole. We think this is unlikely however, because we found that GABA_B_ expression is the highest in the apical dendritic layers of ventral CA1 neurons compared with their dorsal counterparts. Activation of postsynaptic neuronal GABA_B_ receptors (Bettler, Kaupmann, Mosbacher, & Gassmann, 2004) was shown to directly inhibit several types of voltage‐sensitive calcium channels and generate inhibitory postsynaptic potentials via G protein‐coupled inwardly rectifying K^+^ (GIRK) channels (Chalifoux and Carter, 2011; Degro, Kulik, Booker, & Vida, 2015; Liu et al., 2012; Sun and Wu, 2009; Yang, Tadavarty, Xu, & Sastry, 2010). This suggests their greater contribution to a hyperpolarizing action of GABA_A_ receptors, specifically in the VH. In turn, this would be expected to lead to a stronger inhibitory control and thus, lower excitability of the ventral CA1 principal cells.

In line with this interpretation, we showed that pyramidal neurons of the ventral CA1 responded with fewer action potentials, following current injections, than neurons in the dorsal and intermediate subdivisions. This finding is in agreement with the work of Maggio and Segal (2009b, but see also: Dougherty et al., 2012; Malik, Dougherty, Parikh, Byrne, & Johnston, 2016) that reported a higher action potential firing frequency in the dorsal, as opposed to the ventral, CA1 neurons. Furthermore, we detected differences in the resting membrane properties of pyramidal neurons that could partly account for the observed differences in the firing frequency‐to‐current relationship. They included: (a) a more depolarized threshold for the generation of the action potential in the ventral subdivision, despite the fact that the RMP was equivalent among all three hippocampal parts; (b) a deeper AHP, that prolonged the neuronal refractory period; and (c) a reduced action potential amplitude in the ventral pyramidal cells, compared with the dorsal and intermediate ones. The latter two effects are likely to depend on the expression and distribution patterns of various sodium and potassium channels (Bean, 2007; Colbert & Pan, 2002). In agreement with this suggestion, a recent study showed that pyramidal neurons of the ventral CA1 region express significantly higher levels of Ca^2+^‐activated SK‐type K^+^ channels. These, in turn, were shown to inhibit NMDAR‐dependent EPSP amplification to a greater degree in the ventral as opposed to the DH (Babiec, Jami, Guglietta, Chen, & O'Dell, 2017).

Neurotransmitter release properties were also altered along the dorsoventral axis. We saw stronger paired pulse facilitation in the dorsal CA1 following stimulation at intervals of 20, 25, and 50 ms compared with the ventral and intermediate parts. These findings align with reports by others for both the CA1 (Papatheodropoulos and Kostopoulos, 2002) and CA3 regions of the hippocampus (Pofantis, Georgopoulos, Petrides, & Papatheodoropoulos, 2015) and suggest that the intermediate and ventral hippocampus display a higher release probability (Zucker and Regehr, 2002). GABAergic modulation influences paired‐pulse facilitation by means of transient depression of postsynaptic inhibition (Davies, Davies, & Collingridge, 1990; Nathan and Lambert, 1991), albeit at IPIs of 100 ms or more. This would be expected to affect GABA_B_ receptors (Gassmann and Bettler, 2012). At least for the dentate gyrus, paired‐pulse responses that are elicited in the 10–40 ms range are modulated by GABA_A_ receptors (DiScenna & Teyler, 1994; Halasy & Somogyi, 1993; Moser, 1996). However, GABA_B_ receptors also modulate the effectivity of GABA_A_ receptors (Bettler et al., 2004). The relatively higher levels of GABA_B_ receptors and lower levels of GABA_A_ receptors in the ventral compared with the dorsal CA1 regions that we detected in this study, suggest that these receptors may comprise the main mechanism through which paired pulse responses differ along the dorsoventral axis.

Inhibition in the hippocampus is not only mediated by GABA receptors. MGlu1 also contributes to inhibitory neuromodulation (Ferraguti et al., 2004). In this study, we detected higher levels of mGlu1 receptors in the apical dendrites of the ventral pole compared with the intermediate and dorsal parts. Here, given the predominant localization of mGlu1 receptors on interneurons (Ferraguti et al., 2004), the relative inhibitory control can be expected to be even stronger in the ventral CA1 region. In case of the intermediate CA1, the neuronal excitability that is created by differences in GluN1/GluN2A and GluN1/GluN2B expression is also counterbalanced by a strong GABA_A_‐mediated inhibition and an intermediate GABA_B_ expression, suggesting that strong inhibitory control occurs at this site as well. Furthermore, we detected higher mGlu2/3 expression in the ventral and intermediate subdivisions. These receptors act as inhibitory autoreceptors at glutamatergic terminals (Ferraguti and Shigemoto, 2006). This suggests that a faster reduction of glutamate levels following synaptic activity that would further contribute to reduced excitability in the intermediate and ventral subdivisions, compared with the dorsal pole. These differences are likely to underline the weaker LTP elicited in these subdivisions compared with the dorsal CA1 region.

### 4.2 Expression profiles of the plasticity‐related receptors and their relation to synaptic plasticity

Both dorsal and ventral CA1 expressed persistent LTD of comparable magnitude, whereas the intermediate CA1 expressed only STD. This finding is in accordance with that of Maggio and Segal (2009a) who reported equivalent LTD between the dorsal and ventral subdivisions. Weaker LTD responses have also been reported for the intermediate dentate gyrus, compared with the dorsal dentate gyrus, *in vivo* (Kenney and Manahan‐Vaughan, 2013a, 2013b). Functionally, the preference of the IH for short‐term changes in synaptic weights may reflect its postulated role in rapid learning and the updating of already stored information (Bast et al., 2009). MGlu2/3 receptors are specifically involved in the expression of LTD (Altinbilek & Manahan‐Vaughan, 2009; Manahan‐Vaughan 1997) and NMDAR are involved in dorsal CA1 LTD (Manahan‐Vaughan, 1997) and intermediate dentate gyrus LTD (Kenney and Manahan‐Vaughan, 2013a, 2013b). In the ventral CA1, we detected the highest levels of GluN2B and mGlu2/3 expression, with lowest levels being evident in the dorsal CA1. The expression of these receptors in the intermediate CA1 was on a ‘sliding scale’ between these extremes. In the dorsal CA1 region, strong NMDAR activation results in LTP, whereas weaker activation results in LTD (Cummings, Mulkey, Nicoll, & Malenka, 1996) and inadequate mGlu2/3 activation results in a curtailed expression of LTD (Manahan‐Vaughan 1997). Thus, the precise levels of expression and/or subunit constellations of NMDAR coupled with expression levels for mGlu2/3 in the intermediate CA1 may underlie the STD effects we observed. Thus interpretation, in turn, suggests that the LTD elicited in the ventral CA1 may be mechanistically different to that elicited in the dorsal pole.

We showed that LTP was successfully elicited in all three subdivisions of the hippocampal longitudinal axis. The DH exhibited significantly greater potentiation compared with the ventral and intermediate subdivisions, however, with the latter two subdivisions showing similar response profiles. This finding is in line with existing reports of greater LTP in the dorsal CA1 as opposed to its ventral counterpart (Maggio and Segal, 2007a, 2007b; Papatheodoropoulos and Kostopoulos, 2000). Mechanistically, such differences, in the magnitude of potentiation, may be related to the differential expression of plasticity‐related receptors that we detected along the dorsoventral hippocampal axis as well as to reported differences in ion channel expression. *In vivo*, GluN2A‐containing NMDARs are important for the induction of the early phase of LTP (E‐LTP, < 1 h), whereas activation of GluN1/GluN2B‐containing NMDARs appear more important for LTP that lasts for longer periods (Ballesteros, Buschler, Köhr, & Manahan‐Vaughan, 2016). GluN1/GluN2B‐containing receptors also require a higher membrane depolarization for their activation compared with GluN1/GluN2A‐containing receptors (Clarke, Glasgoq, & Johnson, 2013). Thus, although GluN2A expression was equivalent across the dorsal, intermediate and ventral CA1 regions, the graded expression of GluN2B‐containing NMDARs can be expected to impact on LTP responses, as it will serve to alter the GluNA/GluN2B ratios across the dorsoventral axis. Additionally, an enhanced SK‐type K+ channel‐dependent suppression of NMDAR activation in ventral CA1 pyramidal cells, as opposed to dorsal CA1 pyramidal cells (Babiec et al., 2017), would be expected to escalate the propensity differences of hippocampal parts in expressing long‐term synaptic potentiation. LTP maintenance is supported by mGlu5 (Mukherjee and Manahan‐Vaughan, 2013) and this receptor also potentiates NMDAR currents (Doherty, Palmer, Henley, Collingridge, & Jane, 1997; Perroy et al., 2008). We found that mGlu5 was equivalently expressed across the hippocampal dorsoventral axis, however, suggesting that this receptor contributes little to the differences in LTP that we observed.

One interesting prediction that arises from the findings of this study is that the weaker LTP levels we observed seem to relate to a putatively stronger inhibitory control within the ventral pole of the hippocampus, which may not necessarily arise as a result of differences in GABAergic receptor expression, but rather may occur due to a more complex interaction between GABAergic, glutamatergic, and dopaminergic receptors, as well as ion channels. This suggests that information encoding by means of LTP does not readily occur in this structure. The higher levels of GluN1/GluN2B receptors suggest however, that under circumstances where this inhibitory control can be overcome, LTP will not only be greater in magnitude but more robust in its persistency. Assuming, in turn, that the VH processes the emotional context of memory (Segal et al., 2010; Strange et al., 2014) and that this is enabled by means of LTP (Whitlock, Heynen, Shuler, & Bear, 2006), this would suggest that strongly salient (emotional) experiences will result in quite robust and persistent encoding in this part of the hippocampus.

## 5 CONCLUSIONS

**Figure 5:**
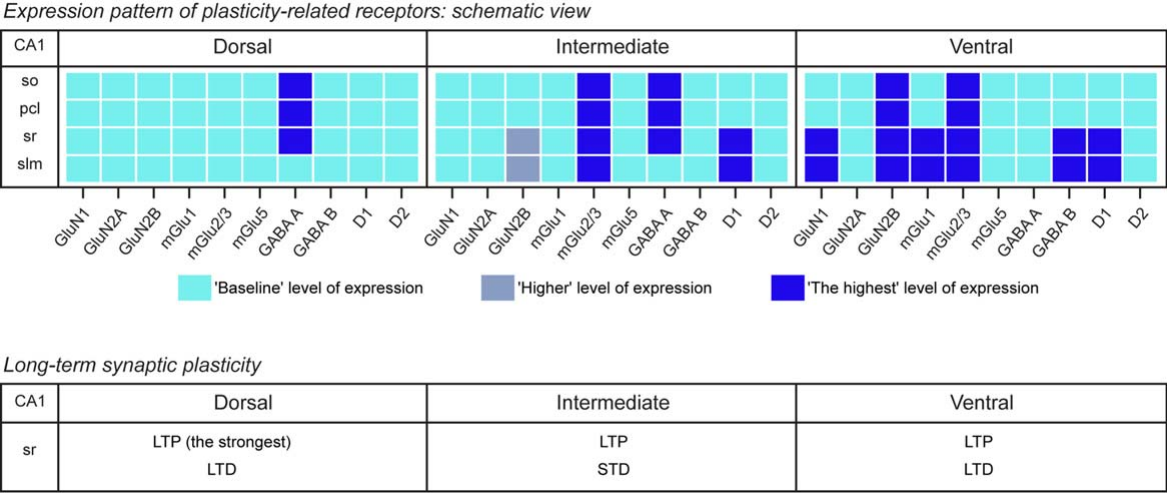
Overview of the hippocampal dorso‐ventral CA1 region differences in receptor protein expression and synaptic plasticity levels. For all receptors, the “Baseline” level of expression refers to the lowest measured level of protein expression; a “Higher” level of expression refers to a significantly higher level of expression compared with the “Baseline” level; and the “Highest” level of expression corresponds to a significantly higher level of expression compared with the “Baseline” and “Higher” levels. pcl, pyramidal cell layer; so, Stratum oriens; sr, Stratum radiatum; slm, Stratum lacunosum‐moleculare [Color figure can be viewed at wileyonlinelibrary.com]

In summary, this study demonstrates that GluN1, GluN2B, GABA_A_, and GABA_B_ receptors, mGlu1, mGlu2/3, and dopamine D1 receptors are heterogeneously distributed across the dorsoventral CA1 region. Strikingly however, these differences take the form of a gradient whereby, GluN1, GluN2B, mGlu1, GABA_B_, and D1‐receptors are expressed lowest in the dorsal CA1 and highest in the ventral CA1, whereas GABA_A_ is expressed highest in the dorsal CA1 and lowest in the ventral CA1. Differences in neuronal excitability, neurotransmitter release probability and synaptic plasticity appear, along with these expression gradients (Figure 5). Taken together, these findings suggest that differences in the expression levels of plasticity‐related receptors underlie functional distinctions in synaptic information storage along the dorsoventral hippocampal axis. These, in turn, may underlie the ascribed role of the different subdivisions of the dorsoventral hippocampal axis in information processing, learning and memory.

## Supporting information

Additional Supporting Information may be found online in the supporting information tab for this article.

Supporting Information Figure 1

Supporting Information Figure 2

Supporting Information Figure 3
