# Supplementary figures and images for: Less means more: The magnitude of synaptic plasticity along the hippocampal dorso‐ventral axis is inversely related to the expression levels of plasticity‐related neurotransmitter receptors
Source: Hippocampus. 2017 Dec 11;28(2):136–50. doi: 10.1002/hipo.22816 (PMC5814924; doi:10.1002/hipo.22816)

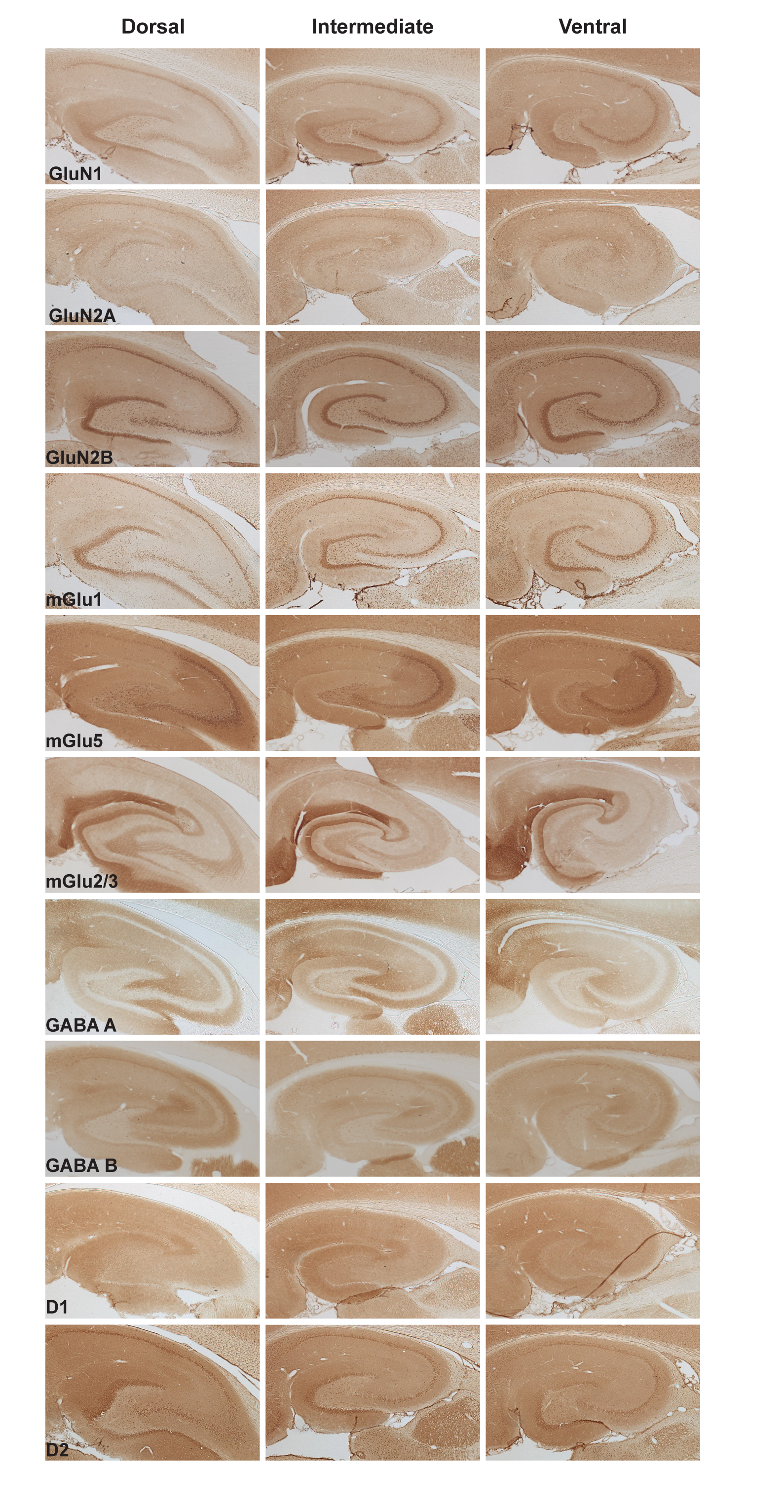

Supplement: Supplementary file 1 — Supporting Information Figure 1 [file HIPO-28-136-s001.tif]

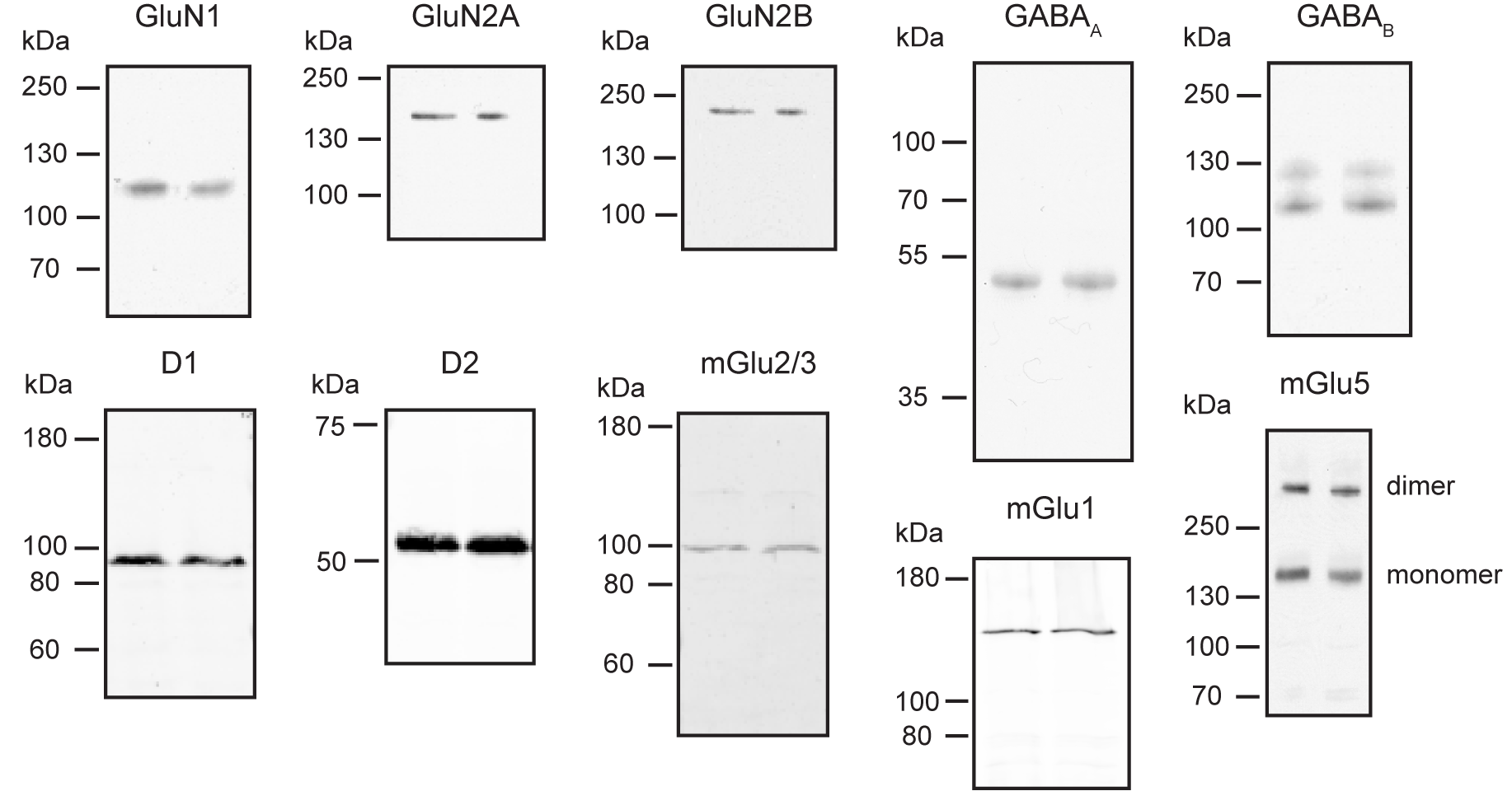

Supplement: Supplementary file 2 — Supporting Information Figure 2 [file HIPO-28-136-s002.tif]

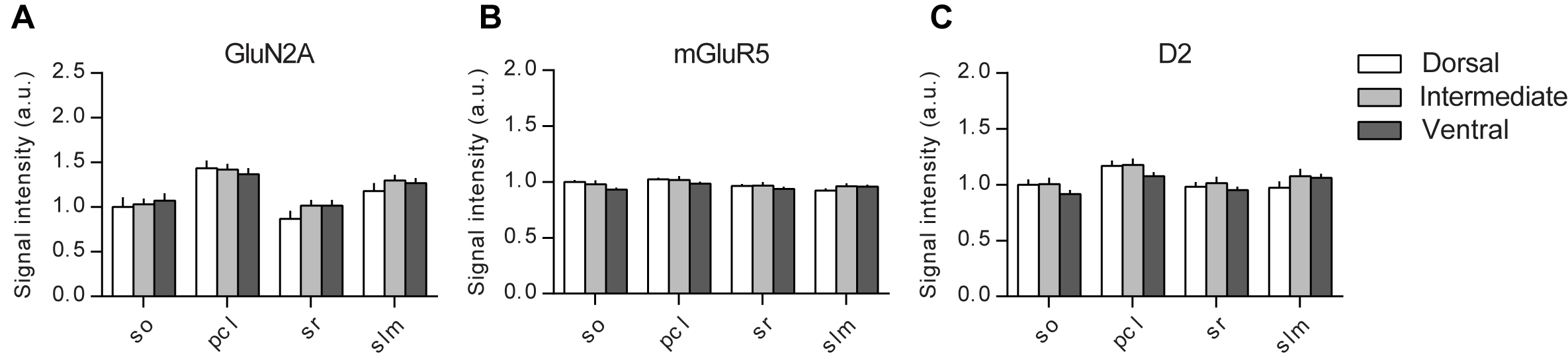

Supplement: Supplementary file 3 — Supporting Information Figure 3 [file HIPO-28-136-s003.tif]
